# Optical Coherence Tomography Velocimetry for In-Line Processing: The Spherical-to-Wormlike Micelle Transition

**DOI:** 10.1021/acsengineeringau.5c00045

**Published:** 2025-11-19

**Authors:** Owen Watts Moore, Thomas Andrew Waigh, Philip Martin, Cesar Mendoza, Harvey Brimelow, John Naughton, Adam Kowalski

**Affiliations:** † Biological Physics, Department of Physics and Astronomy, 5292 The University of Manchester, Manchester M13 9PL, United Kingdom; ‡ Department of Chemical Engineering, 5292 The University of Manchester, Manchester M13 9PL, United Kingdom; ¶ Unilever Research & Development, Port Sunlight Laboratory, Quarry Road East, Bebington, Wirral CH63 3JW, United Kingdom; § Photon Science Institute, 5292 The University of Manchester, Manchester M13 9PL, United Kingdom

**Keywords:** optical coherence tomography, velocimetry, surfactants, in-line processing, wormlike micelles, rheology

## Abstract

Personal care products are often dynamically formulated *in situ*. Variations in the chemistry of the base components (e.g., their polydispersities or ionic contents) require extensive off-line rheological analysis to ensure the products meet benchmarks for performance and thus consumer satisfaction. An in-line alternative for rheological quality control and monitoring thus has the potential to improve efficiency on industrial pipelines. Therefore, we demonstrate optical coherence tomography velocimetry (OCT-V) for the in-line processing of a shampoo based on SLES and CAPB with varying concentrations of salt. OCT-V is a noninvasive quasi-elastic light scattering technique, capable of spatially resolved velocity measurements with an axial depth resolution of 9 μm and a penetration depth of 1.5 mm into the samples. Our in-line apparatus uses infrared light (the wavelength is 1315 nm) and has been optimized for live manufacturing with a 200 L test rig at the Unilever R*&*D lab. The addition of salt to the shampoo is used for *in situ* formulation to increase the viscosity, inducing the spherical-to-wormlike micelle transition. To create in-line rheological benchmarks, we measured time averaged velocity profiles and transient velocity fluctuations in the shampoo formulations at imposed flow rates of *Q* = 500 and 1000 L/h. For a shampoo formulation with 1.1*%* NaCl salt, fits of the Hagen–Poiseuille equation to velocity profile data give flow rates that are in close agreement with the imposed values, *Q*_*HP*_ = 502 ± 2 L/h and 944 ± 4 L/h, corresponding to a 0.4*%* and 5.6*%* error, respectively. Combined with measurement of the longitudinal pressure drop, this could be used to calculate constitutive properties, such as the viscosity. The standard deviation of the distribution of transient velocity fluctuations is a decreasing function of salt concentration due to the increasing viscosity. If calibrated to the desired end product, the velocity fluctuations could also be used as a reproducible indicator of product quality on industrial pipelines.

## Graphical abstract

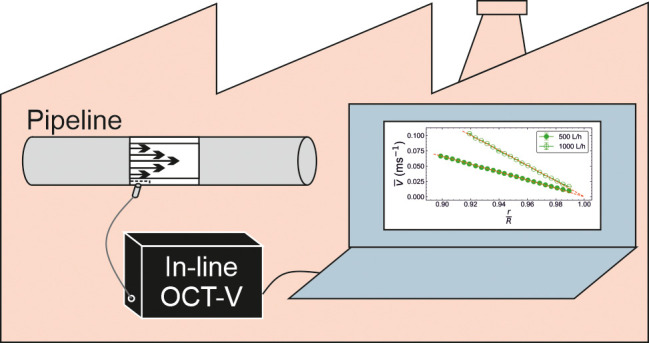

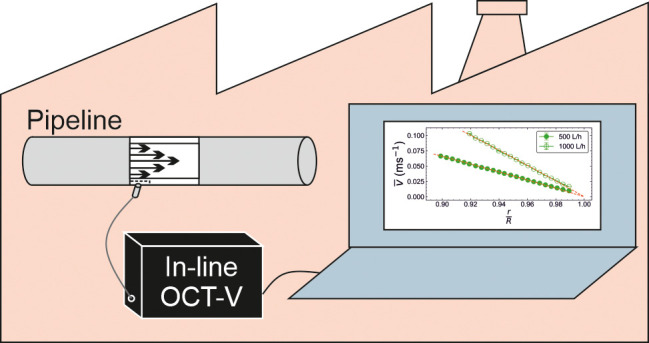

## 1 Introduction

Surfactants form the basis of many personal care products, such as shampoos, performing a number of functions including cleaning, foaming, and the stabilization of additives such as fragrances.^1,2^ Surfactants spontaneously self-assemble into micellar structures in aqueous solution^3,4^ with the bulk rheology strongly dependent on the particular micellar morphology chosen.^5–8^ The morphology is dependent on the surfactant packing parameter, *P*_*c*_, given by the ratio of the effective packing area of the hydrophobic tail group to the hydrophilic headgroup.^3^ Transitions between different morphologies can be induced by altering *P*_*c*_ through changes to surfactant concentration, pH, temperature, or, for ionic surfactants, the addition of salt to screen the repulsive forces between the charged head groups.^4,5,9–11^ The ability to fine-tune surfactant solutions in this way is beneficial to manufacturers as the bulk mechanical properties of personal care products are important for performance and thus consumer satisfaction.^2^

Sodium laureth sulfate (SLES) is a surfactant commonly found in personal care products due to its strong cleaning and foaming properties and mildness to the skin.^2^ Its anionic headgroup means the addition of salt ions alter its rheology.^11^ The distribution of the lengths of the hydrophobic tails in SLES and other surfactant molecules can vary between suppliers, introducing variability into the product quality.^12^ To prevent this, samples must be taken at several points in the formulation process and analyzed *ex situ* to ensure the product meets rheological benchmarks. An *in situ* method for dynamically determining rheological behavior is thus an attractive prospect, promising large improvements in quality control, efficiency, costs and wastage.

In this letter, we present the first application of optical coherence tomography velocimetry (OCT-V) in a real factory setting, at the Unilever R*&*D facility, to characterize differences between shampoo formulations based on SLES and cocamidopropyl betaine (CAPB) with varying salt concentrations during live manufacturing. We have optimized our in-line OCT-V apparatus, previously used to characterize the dynamic behavior of a lamellar gel network (e.g., a hair conditioner) and a solution of milk powder in water on a lab-based test rig,^13^ for use in an industrial environment. We first show time averaged velocity profiles, giving insight into the fluid’s bulk rheological properties before analyzing the transient velocity fluctuations, revealing evidence for the spherical-to-wormlike micelle transition. The ability to differentiate between similar fluids with these measurements suggests that they could be used to create fluid-specific benchmarks for quality control that could be used by process engineers with a wide range of applications for industrial fluids.

OCT-V functions well in experiments with a lab-based rheometer using DNA solutions,^14^ model hard colloidal spheres,^15,16^ solutions of polyacrylamide,^17^ tomato ketchup,^18^ shampoo,^18^ margarine,^15^ chocolate,^18^ starch^19^ and lamellar gel networks.^20^ Unlike similar velocimetric light scattering techniques, such as photon correlation spectroscopy and laser Doppler velocimetry, OCT-V can be used with opaque fluids without the need to seed the fluid with tracer particles. Ultrasonic velocimetry has been used extensively as an in-line probe for opaque fluids.^21^ However, in order to achieve the acoustic contrast necessary for a signal, the particles in the fluid must be no smaller than 1/4 – 1/2 of the ultrasound wavelength.^22^ In a high frequency experiment (∼ 8 MHz) for an aqueous suspension, this would limit the particle size to ≥ 50 μm. Ultrasound methods are also sensitive to particle concentration and temperature variations, which can significantly affect the speed of sound in a fluid.^22^ In contrast, OCT-V only requires the fluid be sufficiently opaque to produce a speckle pattern,^23^ resulting in a broader range of potential in-line applications, such as for nanostructured colloidal fluids.

## 2 Experimental Methods

### 2.1 Optical Coherence Tomography Velocimetry

OCT was first developed as an imaging technique in the early 1990s.^24^ Since then, most of its applications have been medical, including imaging of soft tissue and the eye.^25^ OCT was subsequently adapted to measure the Doppler shift of the light due to a moving sample.^26–28^ As with the imaging technique, this has mostly been used in medical contexts, such as measuring blood flow in capillaries.^29^ However, in the past 15 years Doppler based OCT has increasingly been used to study the rheology of complex fluids.^15,30–32^

Most OCT apparatus now function in the frequency-domain due to improved imaging rates and reduced mechanical noise.^23,33,34^ However, our apparatus is unusual in that it functions in the time-domain due to the superior dynamic range for velocity measurements and relative simplicity of the detection system. In our case, the raw signal is a time varying interference pattern with a frequency equal to the Doppler shift in the light due to the moving sample. The Doppler frequency can then be extracted via a Gaussian fit to the power spectral density (PSD) of the time-domain signal, and the velocity can be calculated via the equation^15^

$$v=\frac{\lambda_{0}f_{D}}{2\sin\left(\theta_{a}\right)}$$ 1

where λ_0_ is the light source central wavelength, *f*_*D*_ is the Doppler frequency, and θ_*a*_ is the angle of the beam relative to the normal of the fluid surface. A sequence of such measurements can be made at a point in the flow to produce either time-averaged or time-series velocity data.

The OCT-V apparatus used in this study is an iteration of that described in Watts Moore et al. (2025).^13^ The key differences from the previous design center around improving portability and safety for use in an industrial environment. The superluminescent diode light source has been upgraded to a Thorlabs SLD1310 device with a central wavelength of 1315 nm, a bandwidth of 90 nm, and a maximum optical power of 30 mW. It is housed in a Thorlbas CLD1015 compact laser diode/temperature controller mount to reduce the apparatus footprint. A Thorlabs BXC21 boxed optical coupler with a 99:1 splitting ratio is used to send 99*%* of the source light to the sample in order to reduce the need for attenuation in the reference arm and reduce the number of loose optical fibers. However, the signal was still found to be at a maximum when a 20 dB (increased from 15 dB) attenuator was used in the reference arm of the interferometer. The full apparatus is contained in a box with a self-contained power supply, protecting the apparatus from potential environmental hazards. The light source, scanning mirror, and data acquisition are operated via a wired connection from a laptop running a LabVIEW control program. The axial depth resolution of the apparatus is 9 μm, allowing spatially resolved measurements of the velocity to be made in 3.4 pL volumes of fluid. The maximum probing depth of an OCT measurement is limited by the effects of multiple scattering to be ∼ 1.5 mm. This is dependent on the optical properties of the sample including scattering anisotropy and mean scattering free path, so calculations of the maximum probing depth can be complex.^23^ We have conducted OCT-V experiments with similar fluids in a shear rheometer and found the measurements agree well with the velocity of the upper plate i.e. a direct mechanical calibrant was available. Since OCT-V cannot penetrate across the whole pipe diameter, the maximum probing depth was determined by the ability to resolve a Doppler peak in the PSD and our experience with using OCT-V with the rheometer.

The probe consists of a perspex section of pipe with screw fittings on either end and the OCT optics was attached to the exterior. This allowed both the beam to enter the flow and the probe to be conveniently inserted into pre-existing pipelines. The modular design separates our apparatus from those used in similar studies based on commercial frequency-domain OCT apparatuses,^35–37^ allowing it to be used in an industrial setting. The measurement position was 25 cm downstream of the outlet of a 90° pipe bend. The velocity profile measurements were made by taking data at each position for 10 s. Each experiment was repeated three times, with the mean calculated. Measurements of the transient velocity were made at a radial position 635 μm from the pipe wall for 300 s. The velocity fluctuations were then calculated using the formula $\frac{v-\mathrm{v̅}}{\mathrm{v̅}}$ , where *v* = *v*(*t*) is the time-resolved velocity and *v̅* is the time averaged mean velocity. To get an accurate position measurement, the refractive index, *n*, of the fluid must be known. To estimate this, we used an off-line OCT apparatus combined with a rheometer to measure the known size of the rheometer gap.^20^ This gives *n* = 1.47 and *n* = 1.52 for the 0*%* and 1.1*%* salt formulations respectively at the near-infrared wavelength used (1315 nm). A schematic diagram of the OCT-V apparatus and probe is shown in Figure 1.

**Figure 1:**
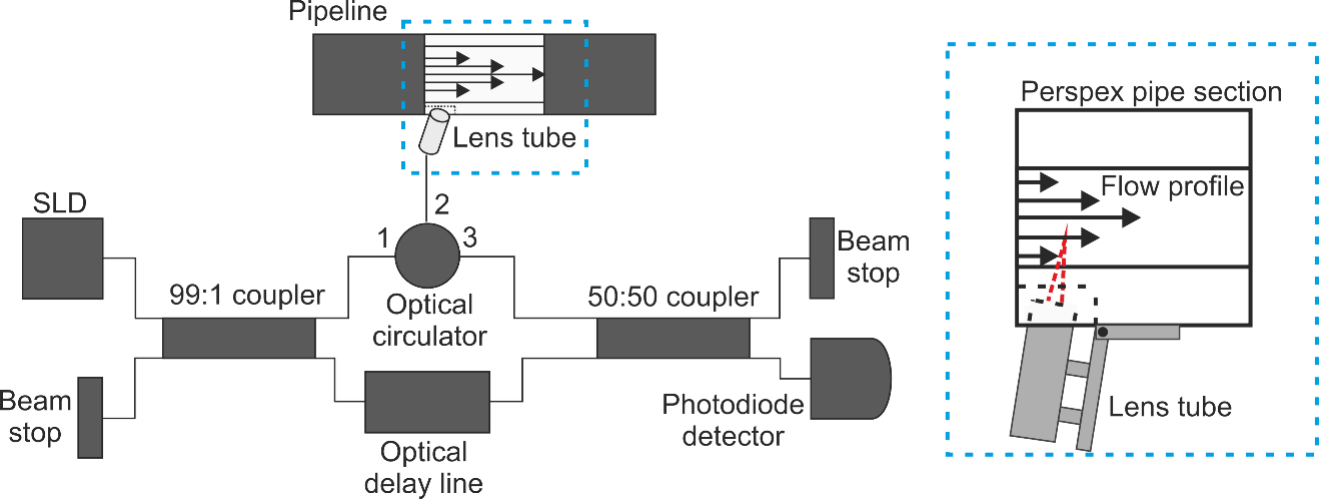
Schematic diagram of the in-line OCT-V apparatus. The infrared superluminescent diode (with a wavelength of 1315 nm) light source is connected to each optical component via single mode optical fibers. The perspex section of pipe allows entry of the light into the flow and is highlighted with dashed lines.

### 2.2 Pipeline

The pipeline consists of 1.5” stainless steel ILC dairy pipe connecting a 200 L mixing vessel, an Alfa Laval SRU rotary lobe pump, and a Silverson 275/400 multistage high shear mixer. The OCT measurements are made 25 cm downstream of a 90° pipe bend exiting the Silverson mixer which is used during key manufacturing steps but is turned off during OCT measurement. This setup replicates the conditions in Unilever manufacturing plants. The internal diameter of the ILC pipes is 34.5 mm. However, due to design constraints, the internal diameter of our OCT probe was 32 mm. A simplified schematic diagram is shown in Figure 2.

**Figure 2:**
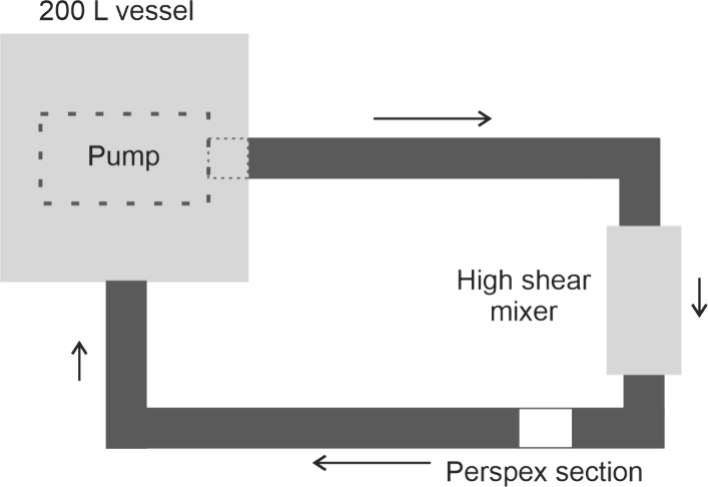
Bird’s-eye view of a schematic diagram of the pipeline on which the OCT-V measurements were made. The pump sits beneath the 200 L mixing vessel. The flow was circulated through the 1.5 inch stainless steel ILC pipes via a high shear mixer. OCT measurements were taken in the perspex section.

### 2.3 Materials

Two slightly different formulations of the base fluid were used in this paper, each made *in situ* to mimic industrial shampoo manufacturing conditions. The first was used in all of the OCT-V experiments and the rheometry experiments with 1.1*%* NaCl salt. The total batch had a weight of 200 kg with 17.1*%* SLES, 5.3*%* CAPB, and 75*%* water. Silicone oil is commonly used in commercial shampoo formulations as a conditioning agent^38,39^ and was added here in the form of an emulsion of high molecular weight polydimethylsiloxane (PDMS) (DOWSIL CE-1788) with a concentration of 2.5*%* to provide the opacity required for OCT-V to function. Two aliquots of crystalline NaCl salt weighing 0.550 and 1.65 kg were added to the mixing vessel in sequence raising the salt concentration to 0.275*%* and then 1.1*%* w/w, respectively. The second formulation was manufactured in the same way, but had 73.4*%* water, 16.8*%* SLES, 5.2*%* CAPB, 4*%* silicone oil, and 0.5*%* preservatives. This formulation was used when testing the OCT-V apparatus on a lab-based test rig. Two aqueous solutions of NaCl were added to this formulation containing 57.9 g of salt and 580.1 g water and 60.5 g salt and 604.8 g water respectively in order to increase the salt concentration to 0.2*%* and 0.4*%* w/w.

### 2.4 Rheometry

A Bohlin Gemini stress-controlled rheometer was used with a parallel plate geometry in all rheometry experiments. Both the upper and lower plates are made from clear perspex, allowing the use of the apparatus in conjunction with an OCT apparatus. The lower plate was held at a temperature of 40 °C to match the manufacturing temperature of the pipeline by a Julabo F25-MV refrigerated circulator supplying water to the plate via pipes. The rheometer gap was held at 500 μm in every run, while a solvent trap was used to reduce the impact of evaporation from the fluid edge.

Oscillatory strain amplitude and frequency sweeps were conducted to probe the linear rheology, while shear ramps were used for nonlinear measurements. Each amplitude sweep had a fixed angular frequency of ω = 10 rad/s, with 10 periods at each strain value, γ. For the 1.1*%* salt formulation, frequency sweeps were conducted at a fixed strain value of γ = 0.1, determined from the amplitude sweep so as to be within the linear viscoelastic region, with measurements conducted over 5 periods at each value of ω. During the shear ramps, the fluids were sheared at each value of *γ̇*, for 300 s before the final measurement of the viscosity was made with a 5 s integration time.

## 3 Results and Discussion

Combinations of SLES and CAPB are known to form both spherical and wormlike micelles, with reversible transitions between the two states possible, induced by altering the concentration of each surfactant or the salt.^11,40,41^ The specific proportions of SLES and CAPB used in this study undergo the spherical to worm-like micelle transition based on the addition of salt.^40^ The linear and nonlinear rheology of fluids can give insight into the underlying microstructure. For example, entangled solutions of giant worm-like micelles have linear rheology in good agreement with a Maxwell model using a single time scale for stress relaxation at low frequencies,^5,42,43^ and the formation of an entangled network is accompanied by a large increase in the zero shear viscosity and viscoelasticity.^6,44^ In the nonlinear regime, entangled solutions of WLMs often show shear thinning and shear banding as the elongated micelles align with the flow.^7,8,43,45–48^

The results of the amplitude (γ) sweeps for each shampoo formulation can be found in Figure 3a, showing the storage and loss moduli, *G′* and *G*″, plotted as a function of the strain, γ. For 0–0.4*%* salt, the data is noisy so it is hard to determine if there is a linear regime for the given strain range. However, the 1.1*%* salt formulation shows a clear linear region up to γ ∼ 1. Figure 3b shows *G′* and *G*″ as a function of frequency (ω) for the 1.1*%* salt formulation.

**Figure 3:**
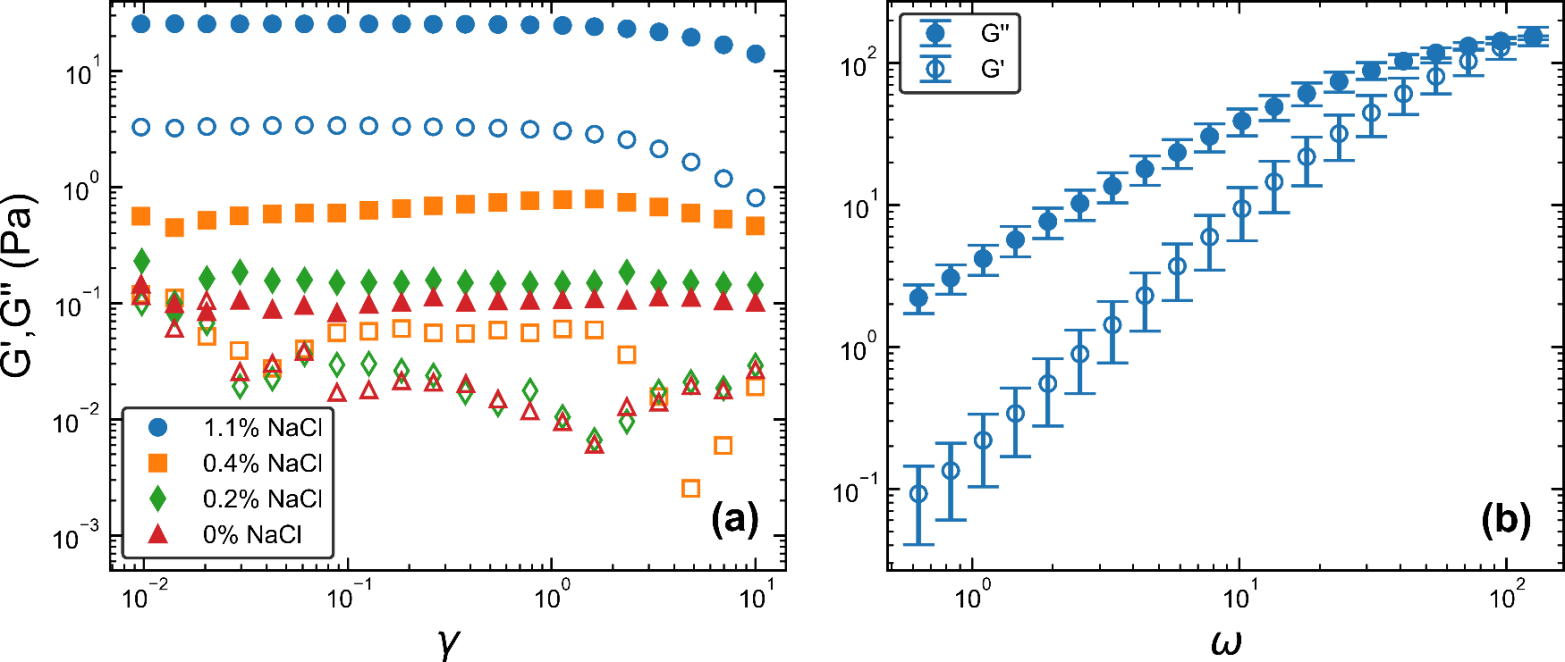
(a) Amplitude sweeps for each salt concentration showing how the storage and loss moduli, *G′* and *G*″, vary with applied strain (γ). Open markers correspond to *G′* and closed to *G*″. (b) Frequency sweep showing the variation in *G′* and *G*″ with angular frequency, ω, for the 1.1*%* salt concentration.

The nonlinear flow curves of the shear stress, τ, as a function of shear rate, *γ̇*, for each shampoo formulation are shown in Figure 4. The stresses of the 0*%*, 0.2*%*, and 0.4*%* salt formulations behave similarly, initially increasing quickly with *γ̇*, before reaching an approximately linear response corresponding to a constant viscosity. In contrast, the 1.1*%* salt formulation shows an approximately linear region up to *γ̇* ≈ 30 – 40 s^–1^, followed by a plateau, with a limited reduction in stress, τ. The viscosity of the 1.1*%* formulation is clearly much larger than the lower salt concentrations over most of the measured range of *γ̇*. A stress plateau in the flow curve is usually indicative of a transition to shear banding.^7,8,43,45–48^ In solutions of WLMs, the plateau exists over a range of shear rates bounded by two critical values, *γ̇*_1_ and *γ̇*_2_. When sheared at a constant rate above *γ̇*_1_, WLMs align with the flow in a locally confined region, producing two bands with distinct shear rates given by *γ̇*_1_ and *γ̇*_2_. This reduces the effective viscosity, causing the stress plateau. As the shear rate is increased, the high shear region grows until it encompasses the whole fluid.^46,49,50^

**Figure 4:**
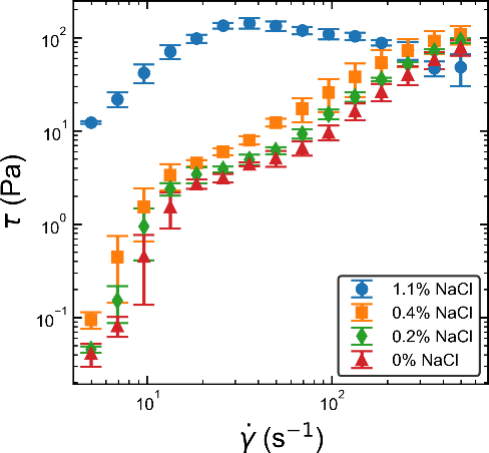
Plot of the shear stress, τ, against the shear rate, *γ̇*, for shampoo formulations with varying salt concentration measured in a fluids shear rheometer.

The large increase in viscosity and the stress plateau seen in the 1.1*%* salt formulation in Figure 4 is typical of the transition from spherical micelles to entangled WLMs that we would expect for this fluid. This is in apparent conflict with the results in Figure 3b, which do not show the characteristic Maxwell-like behavior. This discrepancy is likely explained by the addition of the PDMS emulsion, as the addition of small amounts of high molecular weight polymer to WLMs can alter the linear rheology, causing it to resemble that of a polydisperse polymer solution (containing much shorter chains) rather than a simple single-component Maxwell model due to giant worm-like micelles.^51^

In cylindrical pipes, fluids experience a radial shear gradient, with the maximum value of *γ̇* at the pipe boundary. The velocity profile of a Newtonian fluid in a cylindrical pipe is given by the parabolic Hagen–Poiseuille (HP) equation^52^

$$v\left(r\right)=\frac{\Delta P}{4\mu L}\left(R^{2}-r^{2}\right)$$ 2

where *v* is the flow velocity, *R* is the pipe radius, *r* is the radial position in the pipe, μ is the fluid’s dynamic viscosity, and Δ*P* is the change in pressure over a horizontal length, *L*, of the pipe. If a fluid has nonlinear rheology, the viscosity may not be constant throughout the pipe, changing the shape of the velocity profile. Figure 5a shows the velocity profiles close to the pipe wall taken at imposed flow rates of *Q* = 500 and 1000 L/h for formulations with 0*%*, 0.275*%* and 1.1*%* salt. The velocity profiles for 0*%* and 0.275*%* salt show similar, nonmonotonic behavior. The 1.1*%* salt profiles have a significantly lower gradient, with the *Q* = 1000 L/h profile lining up closely with the *Q* = 500 L/h profiles of the lower salt concentrations, while showing a smooth monotonic increase. To understand this data it is useful to make a rough calculation of the Reynolds number, *Re*, using the formula

$$Re=\frac{\rho VD}{\mu }$$ 3

where ρ is the fluid density, *V* is the average flow wise velocity, and *D* is the pipe diameter. Since each shampoo formulation is mostly water, ρ ≈ 1000 kg/m^3^. A single representative value of μ is difficult to discern from Figure 4, but we can make order of magnitude estimates of μ ∼ 0.1 Pas for the 0*%* and 0.275*%* salt formulations and μ ∼ 1 Pas for the 1.1*%* salt case. When *Q* = 1000 L/h, this gives *Re* ∼ 100 for 0*%* and 0.275*%* salt and *Re* ∼ 10 for 1.1*%* salt. This indicates that the flow should be laminar in all cases if the fluids act in a Newtonian manner e.g. elastic turbulence can be neglected.

**Figure 5:**
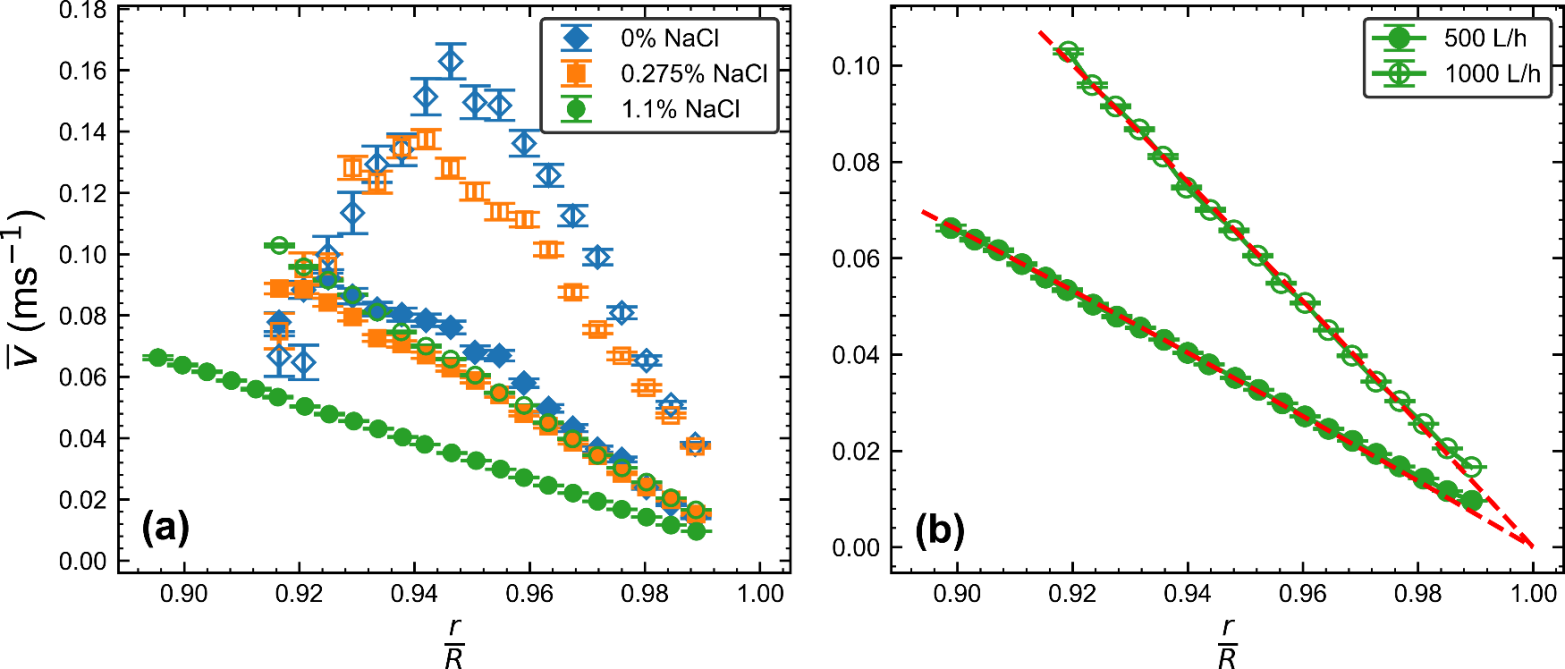
(a) Plots of the time averaged velocity, *v̅*, against the normalized radial position in the pipe, *r*/*R*, for each salt concentration of the surfactants, where *r* is the radial distance of the OCT-V measurements and *R* is the radius of the pipe. Filled markers correspond to *Q* = 500 L/h and open markers to *Q* = 1000 L/h, where *Q* is the flow rate. The peak in the velocity at low salt concentrations is due to vorticity. (b) Velocity profiles for 1.1*%* NaCl formulation with fits of the Hagen–Poiseuille eq (eq 2) overlaid as dashed lines.

These measurements were made at a distance 25 cm downstream from a 90° pipe bend as measured from the bend outlet. Pipe bends introduce vorticity into the flow that can disrupt the downstream laminar radial velocity profile, potentially making it nonmonotonic, as can be seen in Figure 5a for 0*%* and 0.275*%* salt. This was first analytically described by Dean (1927, 1928).^53,54^ However, factors like pipe geometry and pulsatile flow can strongly influence the shape of the downstream velocity profile, making it difficult to predict in imperfect manufacturing conditions.^55,56^ For laminar flow in cylindrical pipes, the entrance length is given by *l*_*e*_ = 0.06*DRe*.^57^ Using our estimates of *Re*, this gives *l*_*e*_ ∼ 20 cm for ≤ 0.275*%* salt and *l*_*e*_ ∼ 2 cm for 1.1*%* salt when *Q* = 1000 L/h. This suggests that it is reasonable to assume that the velocity profile in the 1.1*%* case is fully developed at the point of measurement. However, when the salt concentration is ≤ 0.275*%*, the bend may still be having a significant effect on the velocity profile.

Defining the apparent shear rate, *γ̇*_*app*_, as the maximum shear rate in the pipe for a Newtonian fluid gives *γ̇*_*app*_ = 43.2 s^–1^ and *γ̇*_*app*_ = 86.3 s^–1^ for *Q* = 500 and 1000 L/h, respectively. A linear approximation of the gradient of the 1.1*%* salt profiles gives *γ̇*_*max*_ ≈ 39.9 and 78.5 s^–1^ agreeing fairly well with what would be expected for a Newtonian fluid. Cross referencing these values with Figure 4, the 500 L/h velocity profile should be approximately Newtonian, while the shear rate for the 1000 L/h example enters the shear banding regime. Figure 5b shows a close-up of the 1.1*%* salt data with fits of the HP equation overlaid. From the fit, a value of the flow rate can be calculated by performing a flux integral of eq 2. This gives *Q*_*HP*_ = 502 ± 2 L/h and 944 ± 4 L/h for the 500 L/h and 1000 L/h cases respectively, constituting a 0.4*%* and 5.6*%* error compared with the imposed flow rate, *Q*. The close agreement of the calculated and imposed flow rates suggests that the viscosity has increased enough to dissipate the vorticity from the pipe bend by the point at which the measurements are made, resulting in steady, fully developed velocity profiles. The agreement is particularly close in the 500 L/h case, suggesting the choice of a Newtonian velocity profile is a good one. Shear banding in solutions of WLMs in pipe flow takes the form of a region of high shear rate very close to the wall followed by a return to a parabolic radial velocity profile as you move closer to the pipe center.^58^ We cannot determine the upper bound of the shear banding region from Figure 4, only that *γ̇*_2_ ≳ 500 s^–1^. Since this is an order of magnitude greater than the maximum shear rate in the pipe in the 1000 L/h case, it is safe to assume that the high shear region is small and that a fit of the HP equation to the existing data is a reasonable choice. However, the slightly larger discrepancy between *Q*_*HP*_ and *Q* is likely due to the onset of shear banding, which may well be resolvable if measurements could be made closer to the wall e.g. using an electro-optical modulator with the OCT apparatus.

Though we can qualitatively observe when the viscosity becomes sufficiently large to damp the velocity fluctuations and fully developed velocity profiles form, the vorticity due to the pipe bend makes it difficult to quantitatively track the changes in rheology via the velocity profiles. If there was no vorticity, we would expect the velocity profile of any Newtonian fluid driven at the same flow rate to look identical. The fit of eq 2 to the velocity profiles gives $\frac{\Delta P}{4\mu L}$ as the primary parameter. An additional measurement of Δ*P* would be required to differentiate between the fluids and extract a value of the viscosity, μ. With prior knowledge of the desired fluid rheology, the calculated flow rate could be used as a quantitative rheological benchmark. For example, in the case of a shear banding fluid where the values of *γ̇* in the pipe marginally enter the stress plateau regime, the value *Q*_*HP*_/*Q* could provide a measure of the onset of shear banding and be compared to a benchmark value.

Figure 6a shows the probability density functions (PDFs) of the distributions of the transient velocity fluctuations at a normalized radial position of *r*/R = 0.96 for each salt concentration when *Q* = 1000 L/h. The narrowness of the distribution for the 1.1*%* salt formulation relative to the lower concentrations reiterates the marked differences in rheology seen in Figures 4 and 5. As in the discussion surrounding Figure 5, this can be explained in terms of the increased viscosity due to the formation of giant WLMs damping the velocity fluctuations and reducing the impact of the pipe bend vorticity. The distributions when *Q* = 500 L/h show a similar pattern. The standard deviations of each distribution can be found in Figure 6b. The positive correlation between viscosity and salt concentration is captured in the narrowing distributions of the velocity fluctuations. In this case, the standard deviation of the velocity fluctuations is more sensitive than the velocity profiles at resolving changes to the fluid rheology. While this makes the standard deviation a promising candidate for the creation of a benchmark for quality control, care would need to be taken to account for the effects of the distance of the sensor from a physical perturbation, such as the pipe bend, to assess product quality in a repeatable manner.

**Figure 6:**
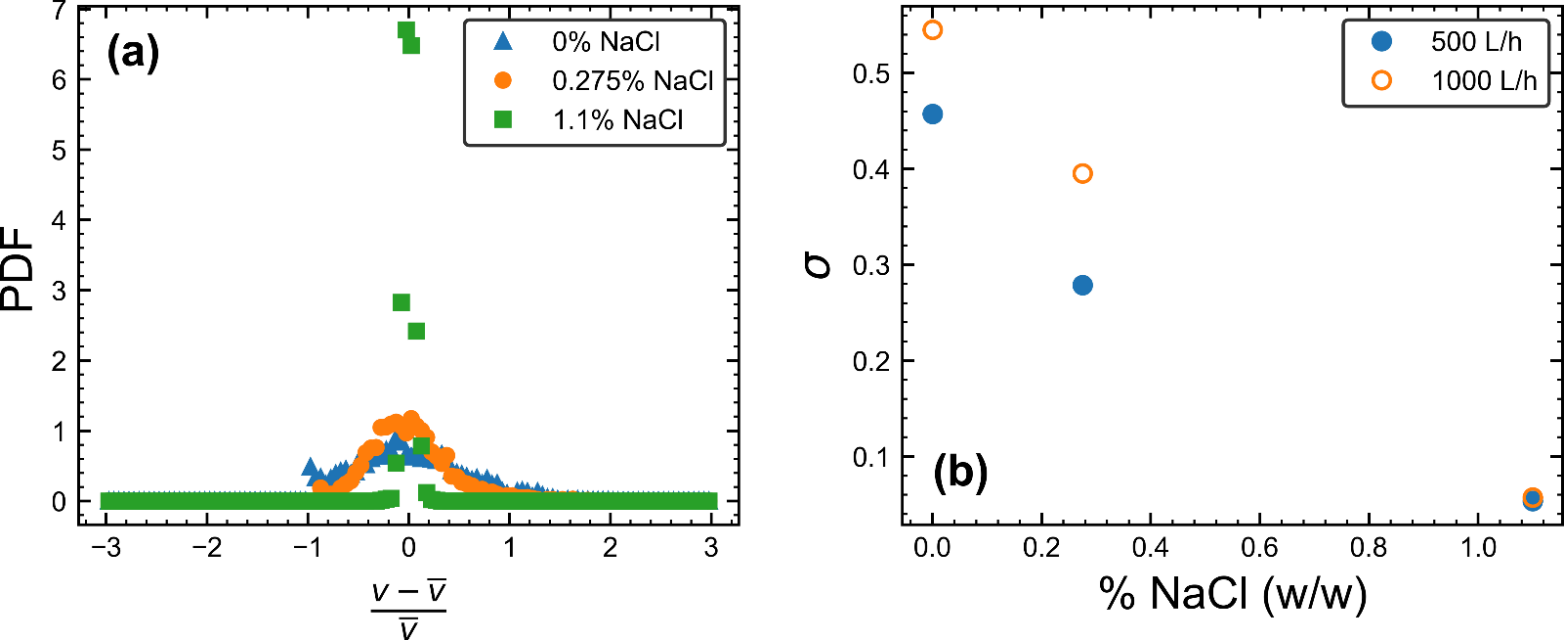
(a) The probability density functions (PDF) of the transient velocity fluctuations, $\frac{v-\mathrm{v̅}}{\mathrm{v̅}}$ , for each salt concentration when *Q* = 1000 L/h, where *v* is the instantaneous velocity, *v̅* is the average velocity and *Q* is the flow rate. (b) Plot of the standard deviation (σ) of the velocity fluctuations as a function of the salt concentration (% NaCl).

The power spectral densities (PSDs) of the transient velocity fluctuations can reveal oscillatory components of the flow, e.g. from positive displacement pumps and information about flow stability.^13^ Figures 7a and 7b show the PSDs for the 0*%* and 0.275*%* salt concentrations when *Q* = 500 L/h. In each, a single well-defined peak can be seen at 5.4 and 5.1 Hz, respectively. This corresponds to the frequency of the pump driving the flow. In the corresponding spectra when *Q* = 1000 L/h, found in Figures 7d and 7e, these peaks disappear as the pump frequency is out of the measurement range. Figure 7c shows the spectrum for the 1.1*%* salt formulation when *Q* = 500 L/h. There are now 4 visible peaks with central frequencies *f*_1_ ≈ 0 Hz, *f*_2_ ≈ 2.45 Hz, *f*_3_ ≈ 3.67 Hz, and *f*_4_ ≈ 4.9 Hz. The peak at *f*_3_ is the largest with nonzero frequency and is the only peak not present in the spectrum for *Q* = 1000 L/h (Figure 7f). In analogy to the behavior of the spectra for the 0*%* and 0.275*%* salt formulations, this suggests that *f*_3_ is the primary pump frequency. Despite the increase in flow rate, the peaks at *f*_1_, *f*_2_, and *f*_4_ all remain present in Figure 7f when *Q* = 1000 L/h. The origins of these peaks are unknown. However, slow oscillations on the time scale of the experiment are visible in the raw data, accounting for the peaks at *f*_1_. The fact that doubling the flow rate does not significantly change the values of *f*_2_ and *f*_4_ indicates they are due to external vibrations. However, there were no obvious changes to the environment that would warrant the onset of an oscillation of similar magnitude to the pump frequency. Since the flow rate, and presumably the pump frequency, doubles, the unchanged frequency of these peaks could be a harmonic effect due to coupling of fluid elasticity to the pulsatile flow driven by the pump. However, more data at a range of flow rates and salt concentrations would be required to explore this further.

**Figure 7:**
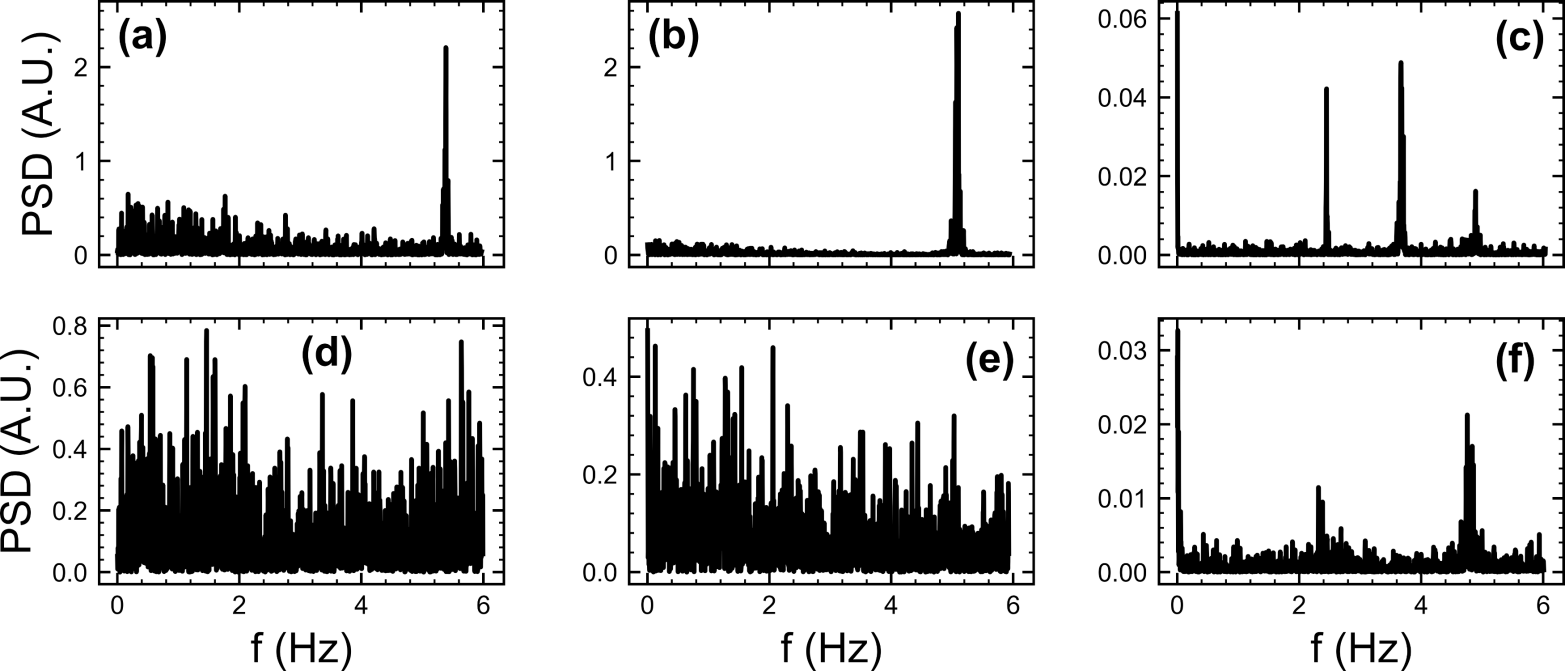
Power spectral density (PSD) plots for the transient velocity fluctuations of the shampoo formulations with 0*%* ((a),(d)), 0.275*%* ((b),(e)), and 1.1*%* ((c),(f)) salt. (a)-(c) correspond to *Q* = 500 L/h, while (d)-(f) correspond to *Q* = 1000 L/h.

In these experiments, it is clear that the position of the sensor relative to the pipe bend has had a significant impact on the results. These effects could be minimized by simply moving the sensor further downstream of the pipe bend. For OCT-V to be used for in-line quality control, an assessment of the effects of physical perturbations on the flow, taking into account viscosity and flow rate, would need to be made on a case-by-case basis. While not necessarily vital, a measurement of the differential pressure would also allow direct quantification of physical properties of the fluid-like viscosity. Improvements to the signal-to-noise ratio of measurements made at low flow rates and close to the pipe wall could be made through the addition of a fiber-based electro-optic modulator (EOM) to the interferometer. For free space implementations of an EOM in lab based OCT-V see refs 14, 16, and 20.

## 4 Conclusions

We have demonstrated the use of in-line OCT-V for monitoring the flow of shampoo formulations based on SLES and CAPB with varying salt concentrations. When the fluid was dominated by spherical micelles at low salt concentrations, a bend in the pipe introduced vorticity into the flow. Though this effect diminishes with distance from the bend, the position of our sensor made measurements of the time-averaged velocity profiles nonmonotonic and it was impossible to quantify the fluid’s physical properties. In future, these measurements could be improved by moving the sensor further downstream from any physical perturbation. Once the salt concentration induced the spherical-to-wormlike micelle transition, the fluid viscosity was increased by at least an order of magnitude, damping the effects of vorticity and allowing fully developed velocity profiles to be measured. These profiles agree well with the imposed flow rate and off-line rheology measurements. As the fluid viscosity increased with salt concentration, the standard deviations of the transient velocity fluctuation distributions decreased due to increased viscous damping. This captures changes to the physical properties of the fluid that proved impossible to quantitatively resolve using velocity profiles. These measurements could form the basis of a set of rheological criteria that would allow the determination of product quality *in situ*.

The application of an improved in-line OCT-V apparatus with a new working fluid (worm-like micelles) and its implementation in a real manufacturing environment are large steps toward a state-of-the-art industrial flow monitoring device. OCT-V has been shown to work with a wide range of fluids across different industries in a lab based rheometer including personal care, food and beverages and biologics.^14–20^ It follows that our in-line device has a broad range of applications, potentially improving efficiency, wastage and quality control by reducing the need for off-line analysis.

## Data Availability

Data will be made available upon request and is hosted at DOI: 10.48420/30344449 via the University of Manchester library.
